# Impaired myocardial perfusion in moderate asymptomatic aortic stenosis relates to longitudinal strain but not non-contrast T1 values

**DOI:** 10.1186/1532-429X-15-S1-O24

**Published:** 2013-01-30

**Authors:** Sacha Bull, Margaret Loudon, Ntobeko Ntusi, Jubin P Joseph, Jane M Francis, Vanessa Ferreira, Stefan K Piechnik, Theodoros Karamitsos, Stefan Neubauer, Saul Myerson

**Affiliations:** 1 OCMR, Oxford, UK

## Background

Myocardial perfusion and strain (in particular longitudinal strain- LS) are reduced in severe aortic stenosis (AS). It is thought that reduced perfusion occurs in the subendocardium secondary to increased LV pressure, leading to fibrosis and reduced LS. This relationship between perfusion, diffuse fibrosis and multidirectional myocardial strain has not been previously investigated in moderate (or severe) AS.

We hypothesized that impaired myocardial perfusion occurs in patients with moderate AS, leading to a degree of diffuse fibrosis (reflected in increased non-contrast T1 values) and impaired longitudinal strain.

## Methods

32 patients with moderate AS (by echo criteria) and 12 age and sex-matched normal controls were recruited. All subjects underwent CMR scanning at 1.5T including valve assessment, stress and rest perfusion, tagging, and non-contrast T1-mapping using the ShMOLLI (Shortened Modified Look-Locker Inversion recovery) sequence, which was previously shown to have a good correlation with histological quantification of diffuse fibrosis. Myocardial perfusion reserve index (MPRI) and strain values were derived from analysis of the perfusion and tagging sequences respectively.

## Results

Despite being well matched for age and sex, there were significant differences between patients with moderate AS and normal controls in longitudinal strain, MPRI and LV mass (p<0.0001, Table 1). There were no significant differences in circumferential strain and T1 values between the two groups. In AS patients there was a moderate correlation with MPRI and LS (r = -0.4, p<0.05), but not circumferential strain (CS). In AS patients T1 values correlated with LV mass (r = 0.4 p<0.01) and peak velocity across the aortic valve (r = 0.5, p< 0.0001) but there was no significant correlation between T1 values and MPRI. These correlations were not seen in the normal controls.

**Table 1:** Patient Characteristics

	**Normal (12)** (12)	Aortic Stenosis (32)	p value
Age	61±5	65±14	0.31
*Sex* (*female/ %*)	3 (25%)	8 (25%)	1
Peak velocity across the aortic valve m/s	-	3.1 ± 0.4	-
*LVM mass (g)*	97 ± 36	147 ± 40	<0.001
*Ejection Fraction (%)*	72 ± 3	74 ± 6	0.6
*T1 values (ms)*	948 ± 14	955 ± 32	0.42
*MPRI*	1.9 ± 0.2	1.3 ± 0.4	<0.001
*Longitudinal strain (%)*	-15 ± 1	-11 ± 2	<0.001
*Circumferential strain (%)*	-18 ± 1	-17 ± 3	0.134

## Conclusions

In moderate AS, there is a moderate correlation between reduced MPRI and impaired longitudinal strain but not circumferential strain. This supports the hypothesis that impairment of myocardial perfusion in AS predominantly affects the subendocardial longitudinal myocardial fibers (rather than the mid-wall circumferential fibers).The exact relationship of perfusion and diffuse fibrosis in moderate AS remains to be fully established however. Non-contrast T1 values (as a surrogate marker for diffuse fibrosis) were not affected by changes in myocardial perfusion but were affected by LV mass in AS patients.

## Funding

This study is funded by the British Heart Foundation, Heart Research UK and the National Institute for Health Oxford Biomedical Research Centre.

